# Supplementary material for: Noise propagation in an integrated model of bacterial gene expression and growth
Source: PLoS Comput Biol. 2018 Oct 5;14(10):e1006386. doi: 10.1371/journal.pcbi.1006386 (PMC6192656; doi:10.1371/journal.pcbi.1006386)
Supplement: S1 Text — Presented in this text are a full derivation of the linear noise model, calculations of statistical properties using Fourier transforms, complete derivations of the results on growth control, detailed calculations of the two-protein an many-protein models, and an analysis of a model where noise is added to ribosomal allocation rather than protein production. (PDF) [file pcbi.1006386.s001.pdf]

Text S1: Supplemental Information for

Noise propagation in an integrated model of bacterial gene expression and growth

ISTVAN T. KLEIJN*, LAURENS H. J. KRAH*, AND RUTGER HERMSEN

Contents

About this document 1

Derivation of the linear noise model 1
- Abundances, volume, and growth rate 1
- Proteome fractions and their dynamics 2
- Stochastic protein-synthesis rate 2
- Stochastic growth 2
- Linearization 3
- Including dynamical gene regulation 4

Calculating statistical properties 4
- Fourier transforms 4
- Statistical quantities 5

Results on growth-control coefficients 7
- Relation between GCCs and FCCs 7
- Sum rules 7

Application to the two-protein toy model 8
- Cross-correlations 8
- Regulation in the two-protein model 8
- Stochastic simulation of the two protein model . . . 9

Application to the many-protein model 9
- GFP reporter fused to the *lac* operon 9
- Parametrization 10
- Alternative choice for abundances and variances . . 11

Alternative model:
Noise in the allocation of the flux 12
- Simulations of the "allocation-noise" model 12

About this document

In this document, we provide detailed derivations and analyses pertaining to the results presented in the main text.

We aim to describe the stochastic behavior of a cell under conditions of steady-state exponential growth. A cell contains many protein species. Each of these may affect growth to some extent via an effect on metabolism. (Note that, here and below, we use the term metabolism in a broad sense; it is intended to encompass all catabolic and anabolic processes required for biomass production and cell growth, including protein synthesis.) As mentioned in the main text, our results are based on several crucial assumptions. In particular, we assume that

- the protein density of the cell is under tight homeostatic control,
- protein synthesis is inherently stochastic,
- the cellular growth rate is an *intensive*[1] quantity.

These assumptions are used repeatedly in the text below.

Derivation of the linear noise model

We start with a detailed derivation of the linear noise model.

Abundances, volume, and growth rate

The abundances of the protein species are given by the vector $\boldsymbol{n}(t) = \{n_1(t), n_2(t), \ldots\}$, where $n_i(t)$ is the copy number of protein species i at time t. We define the total protein mass of the cell by

$$M := \sum_i n_i, \tag{1}$$

where we ignore that different proteins have different molecular weights. (To avoid clutter, we left out the explicit time dependence of the variables; below, we do so whenever this is unlikely to cause confusion.) Under the assumption that the protein density of a cell is tightly controlled, M is proportional to the volume of the cell. Therefore, we define the instantaneous growth rate μ as the rate at which M increases:

$$\mu := \frac{1}{M}\frac{\mathrm{d}M}{\mathrm{d}t}. \tag{2}$$

[1] In this context, an intensive quantity is a quantity that does not change if the size of the cell is changed, provided the concentrations of all enzymes are kept constant. By contrast, an *extensive* quantity is a quantity that, under the same conditions, increases linearly with the size of the cell. It follows that any quantity that is a function of intensive quantities only, is itself an intensive quantity. Also, the ratio of two extensive quantities is an intensive quantity.

Proteome fractions and their dynamics

The proteome fractions of the different protein species in the cell are given by the vector

$$\boldsymbol{\phi} := \frac{\boldsymbol{n}}{M}. \tag{3}$$

Again, assuming that the mass M is proportional to the cellular volume, we can interpret the mass fractions as concentrations.

We denote the rate of protein synthesis by the vector $\boldsymbol{p}(t) = \{p_1(t), p_2(t), \ldots\}$ and assume that, under conditions of balanced exponential growth, degradation of proteins is negligible compared to protein dilution due to growth [40]. Then the time derivative of equation (3) yields the following system of dynamical equations:

$$\frac{\mathrm{d}\boldsymbol{\phi}}{\mathrm{d}t} = \boldsymbol{\pi} - \mu\boldsymbol{\phi}, \tag{4}$$

where the vector $\boldsymbol{\pi} := \boldsymbol{p}/M$ is the protein synthesis rate per unit of protein mass.

By definition, the mass fractions add up to one:

$$\sum_i \phi_i = 1. \tag{5}$$

Therefore, an increase in the concentration of one protein must go at the expense of the concentration of another. Applied to equation (4), this constraint also implies that

$$\mu = \sum_i \pi_i. \tag{6}$$

That is, the growth rate is equal to the total rate of protein synthesis per unit of mass.

In reality, even if protein density is under strict control, it is likely to show fluctuations on short time scales. In mammalian cells, mass fluctuations have indeed been measured, but appeared to dissipate on time scales of less than 20 s [55]. By comparison, the auto-correlation times of the expression and growth-rate fluctuations observed in *E. coli* cells are very long—they are of the order of hours. On this time scale, we therefore expect equation (6) to be a reasonable approximation.

Stochastic protein-synthesis rate

The protein synthesis rate of each protein species i is assumed to have the following form:

$$\pi_i = f_i J(\boldsymbol{n})/M + N_i \tag{7}$$

The first term on the right-hand side models the deterministic dependence of the protein synthesis rate on the protein content of the cell. The function $J(\boldsymbol{n})$ represents the global metabolic flux that fuels protein synthesis and growth. The vector $\boldsymbol{f} = \{f_1, f_2, \ldots\}$ indicates which fraction of this metabolic flux is allocated towards the synthesis of each of the protein species. As such, it obeys the constraint

$$\sum_i f_i = 1. \tag{8}$$

In general, $\boldsymbol{f}$ is itself likely to be a function of intra- and extracellular variables, including the protein content of the cell, due to the dynamical regulation of gene expression. Further below we will reflect on this general case, but, unless indicated otherwise, we focus on the special case where $\boldsymbol{f}$ is constant.

The second term on the right-hand size of equation (7) models the stochasticity of protein synthesis. Each N_i is a colored Ornstein–Uhlenbeck noise source with zero mean, reversion rate β_i, and amplitude θ_i, obeying the following stochastic differential equation (Itō):

$$\mathrm{d}N_i(t) = -\beta_i N_i \,\mathrm{d}t + \theta_i \,\mathrm{d}W_i(t), \tag{9}$$

where each $W_i(t)$ is a Wiener process. Importantly, all noise sources are assumed to be mutually independent.

Stochastic growth

Combined, equations (6) and (7) yield the following expression for the growth rate μ:

$$\mu = J(\boldsymbol{n})/M + \sum_i N_i. \tag{10}$$

The first term, $J(\boldsymbol{n})/M$, is a function of the protein content of the cell; we call it $\mu_\mathrm{d}(\boldsymbol{n})$. The assumption that the growth rate is an intensive quantity translates to the requirement that

$$\mu_\mathrm{d}(\alpha\,\boldsymbol{n}) = \mu_\mathrm{d}(\boldsymbol{n}), \tag{11}$$

for any $\alpha > 0$. Because μ_d is intensive and M is extensive, the metabolic flux $J(\boldsymbol{n}) = M\mu_\mathrm{d}(\boldsymbol{n})$ must be extensive, *i.e.*:

$$J(\alpha\,\boldsymbol{n}) = \alpha J(\boldsymbol{n}), \tag{12}$$

for any $\alpha > 0$. This is indeed routinely assumed in the field of metabolic control analysis [64]. Using equation (11), we note that $\mu_\mathrm{d}(\boldsymbol{n}) = \mu_\mathrm{d}(\boldsymbol{n}/M) = \mu_\mathrm{d}(\boldsymbol{\phi})$, so that equations (7) and (10) can be rewritten as

$$\pi_i = f_i\,\mu_\mathrm{d}(\boldsymbol{\phi}) + N_i, \tag{13}$$

$$\mu = \mu_\mathrm{d}(\boldsymbol{\phi}) + \sum_i N_i. \tag{14}$$

Equation (14) shows that all noise sources affect the growth rate in two ways: directly through the second term, and indirectly through the first, because of fluctuations in the composition $\boldsymbol{\phi}$.

Provided a function $\mu_\mathrm{d}(\boldsymbol{\phi})$ is given, equations (4), (13) and (14) together fully specify the dynamics of $\boldsymbol{\pi}$, $\boldsymbol{\phi}$, and μ.

Linearization

To obtain analytical results, we assumed that fluctuations in the concentrations $\boldsymbol{\phi}$ are small enough to warrant a linear approximation. Below, we describe the linearization of equations (4), (13), and (14) around the time average $\boldsymbol{\phi_0}$ of $\boldsymbol{\phi}$.

We start with equation (4). We introduce transformed variables:

$$\delta\boldsymbol{\phi} := \boldsymbol{\phi} - \boldsymbol{\phi_0}, \tag{15}$$

$$\delta\boldsymbol{\pi} := \boldsymbol{\pi} - \boldsymbol{\pi_0}, \tag{16}$$

$$\delta\mu := \mu - \mu_0, \tag{17}$$

where $\mu_0 := \mu_d(\boldsymbol{\phi_0})$ and $\boldsymbol{\pi_0} := \boldsymbol{f}\mu_0$. In terms of these variables, equation (4) reads:

$$\frac{\delta\dot{\phi}_i}{\mu_0\phi_{0,i}} = \left(\frac{f_i}{\phi_{0,i}} - 1\right) + \left(\frac{f_i}{\phi_{0,i}}\right)\frac{\delta\pi_i}{\pi_{0,i}} - \frac{\delta\mu}{\mu_0} - \frac{\delta\phi_i}{\phi_{0,i}} - \frac{\delta\phi_i}{\phi_{0,i}}\frac{\delta\mu}{\mu_0}. \tag{18}$$

To proceed, we exploit that $f_i = \phi_{0,i}$ to linear order. To see why, we first observe from equation (13) that

$$f_i = \frac{\mathbb{E}[\pi_i]}{\mathbb{E}[\mu_d(\boldsymbol{\phi})]}, \tag{19}$$

where $\mathbb{E}[X]$ denotes the steady-state expectation value of variable X. Next, equation (14) implies that

$$\mathbb{E}[\mu_d(\boldsymbol{\phi})] = \mathbb{E}[\mu], \tag{20}$$

and equation (4) that

$$\mathbb{E}[\pi_i] = \mathbb{E}[\mu\,\phi_i]. \tag{21}$$

Therefore,

$$\frac{f_i}{\phi_{0,i}} = \frac{\mathbb{E}[\mu\,\phi_i]}{\phi_{0,i}\mathbb{E}[\mu]} = 1 + \mathrm{Cov}\left(\frac{\delta\phi_i}{\phi_{0,i}}, \frac{\delta\mu}{\mathbb{E}[\mu]}\right), \tag{22}$$

which is 1 up to quadratic corrections. We can estimate the size of these corrections (the covariance term) as follows. We write:

$$\mathrm{Cov}\left(\frac{\delta\phi_i}{\phi_{0,i}}, \frac{\delta\mu}{\mathbb{E}[\mu]}\right) = R\,\eta_{\phi_i}\eta_\mu, \tag{23}$$

where R is the correlation coefficient between ϕ_i and μ, and η_x is the coefficient of variation of variable x. Taking rather large estimates for each, we arrive at $R\,\eta_{\phi_i}\eta_\mu \lessapprox 0.5 \times 0.4 \times 0.25 = 0.05$. This shows that quadratic corrections are small and we can indeed assume that $f_i = \phi_{0,i}$ to good approximation. (We also verified this using simulations; see below.) Based on this, equation (18) takes the following form after linearization:

$$\frac{\delta\dot{\phi}_i}{\mu_0\phi_{0,i}} = \frac{\delta\pi_i}{\pi_{0,i}} - \frac{\delta\mu}{\mu_0} - \frac{\delta\phi_i}{\phi_{0,i}}. \tag{24}$$

We now move to equation (13). After linearization, it can be written as:

$$\frac{\delta\pi_i}{\pi_{0,i}} = \sum_j C_j^\mu \frac{\delta\phi_j}{\phi_{0,j}} + \nu_i, \tag{25}$$

where we introduce growth-control coefficients (GCCs), defined as

$$C_j^\mu := \left[\frac{\phi_j}{\mu_d}\frac{\partial\mu_d}{\partial\phi_j}\right]_{\boldsymbol{\phi_0}}. \tag{26}$$

(See p. 7 of this text for a further discussion of these quantities.) The Ornstein–Uhlenbeck noise terms $\nu_i := N_i/\pi_{0,i}$ are identical to the original noise terms N_i, except that their noise amplitudes ϑ_i are rescaled as $\vartheta_i = \theta_i/\pi_{0,i}$. Equation (25) shows that the synthesis of a protein species is affected by its own noise source, but also by fluctuations in the concentrations of all other proteins, in as far as these fluctuations affect the growth.

These same methods can be applied to equation (14), the linearized version of which is given by

$$\frac{\delta\mu}{\mu_0} = \sum_i C_i^\mu \frac{\delta\phi_i}{\phi_{0,i}} + \mathcal{N}. \tag{27}$$

Here we introduced the shorthand

$$\mathcal{N} = \sum_i f_i\,\nu_i, \tag{28}$$

which is a weighted sum of all noise sources.

We now use equations (25) and (27) to rewrite equation (24) as

$$\frac{\delta\dot{\phi}_i}{\mu_0\phi_{0,i}} = \nu_i - \mathcal{N} - \frac{\delta\phi_i}{\phi_{0,i}}. \tag{29}$$

Equations (25), (27), and (29) together form the basis for the analyses in the main text.

To test the quality of the analytical results based on the linearization of the equations and the approximation that $f_i = \phi_{0,i}$, we simulated the nonlinear equations (4), (13) and (14) for a system containing just two proteins X and Y, using a convenient nonlinear function $\mu_d(\boldsymbol{\phi}) = (a_X\phi_X + a_Y\phi_Y)/(\phi_X + \phi_Y)$. (Note that μ_d is indeed an intensive function, as it should be. See p. 9 of this document for details on the simulation method.) We then numerically calculated the cross-correlations based on these simulations, and compared them to the corresponding analytical predictions. We chose the parameters of the simulation (including a_X and a_Y) such as to match the parameters used in Fig. 3AB of the main text. In that figure, the simulation results based on the non-linear equations are plotted (gray diamonds) alongside the analytical predictions based on the linearized equations (solid gray line). They show excellent agreement, despite significant noise levels (coefficients of variation of ϕ_X, ϕ_Y, and μ were 0.09, 0.19, and 0.26, respectively). This shows that the approximations made in this section are excellent for sufficiently smooth functions $\mu_d(\boldsymbol{\phi})$.

Including dynamical gene regulation

In the previous sections, we assumed that a fixed fraction f_i of the metabolic flux is allocated towards the synthesis of protein i. In this section we briefly describe the more general case where $\boldsymbol{f}$ depends on the composition of the cell, as expected due to transcriptional and translational regulation. For this case, analytical solutions are generally much harder to obtain. Nevertheless, we can gain some insight into the regulated system by studying the structure of the resulting equations.

We assume that $\boldsymbol{f}$ is an intensive quantity—that is, that it can be written as a function of the relative abundances ϕ. Then equation (13), for the production rate π_i, generalizes to:

$$\pi_i = f_i(\phi)\mu_\mathrm{d}(\phi) + N_i, \tag{30}$$

with $0 \leq f_i(\phi) \leq 1$ and $\sum_i f_i(\phi) = 1$. This expression describes how the composition ϕ of the cell differentially affects the production rate of each protein species. Linearizing equation (30) around $\phi_\mathbf{0}$, we find:

$$\begin{aligned}\delta\pi_i &= \sum_j \frac{\partial}{\partial\phi_j}\left[f_i(\phi)\mu_\mathrm{d}(\phi)\right]\delta\phi_j + N_i \\ &= f_i(\phi_\mathbf{0})\mu_0 \sum_j \left(\frac{\phi_{0,j}}{\mu_0}\frac{\partial\mu_\mathrm{d}}{\partial\phi_j}\right. \\ &\qquad\qquad \left. + \frac{\phi_{0,j}}{f_i(\phi_\mathbf{0})}\frac{\partial f_i}{\partial\phi_j}\right)\frac{\delta\phi_j}{\phi_{0,j}} + N_i, \end{aligned}\tag{31}$$

which results in:

$$\frac{\delta\pi_i}{\pi_{0,i}} = \sum_j \left(C^\mu_j + C^{f_i}_j\right)\frac{\delta\phi_j}{\phi_{0,j}} + \nu_i. \tag{32}$$

Here, new control coefficients emerge: regulational control coefficients (RCCs), defined as:

$$C^{f_i}_j := \left[\frac{\phi_j}{f_i}\frac{\partial f_i}{\partial\phi_j}\right]_{\phi_\mathbf{0}}. \tag{33}$$

These RCCs quantify to what extent small changes in the cell's composition affect the allocation of the metabolic flux over the synthesis of various proteins species. As apparent from equation (32), the RCCs modulate the GCCs in an additive fashion.

Remarkably, the RCCs vanish from the expression for $\delta\mu$:

$$\begin{aligned}\delta\mu &= \sum_i \pi_{0,i}\sum_j\left(C^\mu_j + C^{f_i}_j\right)\frac{\delta\phi_j}{\phi_{0,j}} + \sum_i \pi_{0,i}\nu_i \\ &= \mu_0\sum_j C^\mu_j\frac{\delta\phi_j}{\phi_{0,j}} + \mu_0\sum_{i,j} f_i(\phi_\mathbf{0})C^{f_i}_j\frac{\delta\phi_j}{\phi_{0,j}} + \mu_0\mathcal{N} \\ &= \mu_0\sum_j C^\mu_j\frac{\delta\phi_j}{\phi_{0,j}} + \mu_0\sum_j\left(\sum_i\frac{\partial f_i}{\partial\phi_j}\right)\delta\phi_j + \mu_0\mathcal{N} \\ &= \mu_0\left(\sum_j C^\mu_j\frac{\delta\phi_j}{\phi_{0,j}} + \mathcal{N}\right). \end{aligned}\tag{34}$$

This derivation uses $\pi_{0,i} = \mu_0 f_i(\phi_\mathbf{0})$ and hinges on the fact that $\sum_i f_i(\phi) = 1$, so that $\sum_i \partial f_i/\partial\phi_j = 0$.

The linearized dynamical equation for $\delta\phi_i$ in the presence of regulation becomes:

$$\begin{aligned}\frac{\dot{\delta\phi_i}}{\phi_{0,i}\mu_0} &= \frac{\delta\pi_i}{\pi_{0,i}} - \frac{\delta\mu}{\mu_0} - \frac{\delta\phi_i}{\phi_{0,i}} \\ &= \nu_i - \mathcal{N} - \frac{\delta\phi_i}{\phi_{0,i}} + \sum_j C^{f_i}_j\frac{\delta\phi_j}{\phi_{0,j}}. \end{aligned}\tag{35}$$

By comparing with equation (29) we concluded that gene regulation adds a single term that describes (to linear order) the combined effect on f_i of the fluctuations in the concentrations of all proteins species j.

Calculating statistical properties

Fourier transforms

The linearized expressions from p. 3 enable us to calculate various statistical properties. To do so, it is convenient to work with Fourier-transformed equations and variables. Based on equations (25), (27), and (29), we will now derive expressions for the Fourier transforms of the protein concentrations, the synthesis rates, and the growth rate. Further below, these expressions will be used to compute (co-)variances and correlations between the variables.

We denote the Fourier transform of variable y as $\widetilde{y}$. Taking the Fourier transform of both sides of equation (29) and using the transforms of equations (25) and (27), we find

$$\frac{\widetilde{\delta\phi_j}}{\phi_{0,j}} = \frac{\mu_0}{\mu_0 + \mathring{\imath}\omega}\left(\widetilde{\nu_j} - \widetilde{\mathcal{N}}\right), \tag{36}$$

where ω is the Fourier frequency and $\mathring{\imath}$ denotes the imaginary unit (to distinguish it from indices i). To arrive at this result, we used that

$$\widetilde{\frac{\partial f}{\partial t}} = \mathring{\imath}\omega\widetilde{f} \tag{37}$$

for any time-dependent function f.

By combining equation (36) with the Fourier transform of equation (27), we find a direct expression for the Fourier-transformed growth-rate deviations:

$$\begin{aligned}\frac{\widetilde{\delta\mu}}{\mu_0} &= \sum_j C^\mu_j\frac{\mu_0}{\mu_0 + \mathring{\imath}\omega}\left(\widetilde{\nu_j} - \widetilde{\mathcal{N}}\right) + \widetilde{\mathcal{N}} \\ &= \sum_j\left(\frac{\mu_0}{\mu_0 + \mathring{\imath}\omega}C^\mu_j + \phi_{0,j}\right)\widetilde{\nu_j} \end{aligned}\tag{38}$$

Here we used the definition of $\mathcal{N}$ (equation (28)), the fact that $f_i = \phi_{0,i}$ to linear order, and the sum rule for GCCs presented in the main text:

$$\sum_i C^\mu_i = 0. \tag{39}$$

(See p. 7 for a derivation.) After analogous algebra, equation (25) yields

$$\frac{\widetilde{\delta\pi_i}}{\pi_{0,i}} = \sum_j \frac{\mu_0}{\mu_0 + \mathring{\imath}\omega} C^{\mu}_j \widetilde{\nu_j} + \widetilde{\nu_i}. \tag{40}$$

Statistical quantities

In this section, we first provide analytical expressions for cross-covariance functions between several variables [42]. Equations (36), (38), and (40) have the same mathematical form as analogous equations appearing in earlier models [5, 42]. Consequently, our derivations of the cross-covariance functions are directly parallel to their earlier work. The resulting expressions are then used to calculate various stochastic quantities, including the coefficients of variation of the protein expression levels and the (cross-)correlations between growth rate and expression. Based on these, we provide details on several results presented in the main text, notably the complications in the distinction between intrinsic and extrinsic noise, and the mode decomposition of expression–growth cross-correlation functions.

Definitions and notation

The cross-covariance $\chi_{xy}(\tau)$ between variables $x(t)$ and $y(t)$ is defined as

$$\chi_{xy}(\tau) := \mathbb{E}\left[\delta x(t)\delta y(t+\tau)\right], \tag{41}$$

where $\delta x(t) := x(t) - \mathbb{E}[x]$ and $\delta y(t) := y(t) - \mathbb{E}[y]$. In this notation, a positive value of $\chi_{xy}(\tau)$ means that the value of x co-varies with the value of y taken a time τ later; that is, y "follows" x. The convolution theorem states that

$$\chi_{xy}(\tau) = \int \frac{e^{\mathring{\imath}\omega\tau}\widetilde{\delta x}^{*}\widetilde{\delta y}}{2\pi}\,\mathrm{d}\omega, \tag{42}$$

where the star denotes complex conjugation. The Fourier transforms that were computed in the previous section can therefore be used verbatim to calculate (cross-co)variances for all variables μ, ϕ_i and π_i.

The functions $\chi_{xy}(\tau)$ directly yield various other important quantities. In particular, the variance of x is given by

$$\mathrm{Var}(x) = \chi_{xx}(0), \tag{43}$$

and the coefficient of variation (CV) is defined as

$$\eta_x := \frac{\sqrt{\mathrm{Var}(x)}}{\mathbb{E}[x]} = \frac{\sigma_x}{x_0}, \tag{44}$$

with σ_x the standard deviation of x. The cross-correlation function $R_{xy}(\tau)$ is given by

$$R_{xy}(\tau) = \frac{\chi_{xy}(\tau)}{\sqrt{\mathrm{Var}(x)\mathrm{Var}(y)}}; \tag{45}$$

at delay $\tau = 0$ it reduces to the Pearson correlation coefficient.

The cross-covariances of μ, ϕ_i and π_i are calculated by inserting (36), (38), and (40) into equation (42). In the process, we encounter products of noise sources $\widetilde{\nu_i}^{*}\widetilde{\nu_j}$ (Fourier-transformed cross-covariances of the noise sources), which, based on equation (9), can be expressed as:

$$\widetilde{\nu_i}^{*}\widetilde{\nu_j} = \delta_{ij}\frac{\vartheta_i^2}{\beta_i^2 + \omega^2}. \tag{46}$$

Here, the Kronecker delta δ_{ij} reflects the mutual independence of the noise sources. After using this identity, all cross-covariances can be written as linear combinations of three families of functions, $B_i(\tau)$, $A_i(\tau)$ and $S_i(\tau)$, resulting from three Fourier integrals that can be solved explicitly with Cauchy's residue theorem.

The first function is

$$\begin{aligned} B_i(\tau) &:= \int \frac{e^{\mathring{\imath}\omega\tau}}{2\pi}|\widetilde{\nu_i}|^2\,\mathrm{d}\omega \\ &= \frac{\vartheta_i^2}{2\beta_i}e^{-\beta|\tau|}. \end{aligned} \tag{47}$$

This function is symmetric; it is the auto-covariance of an Ornstein–Uhlenbeck process and therefore characterized by a single timescale β_i.

The second function is

$$\begin{aligned} A_i(\tau) &:= \int \frac{e^{\mathring{\imath}\omega\tau}}{2\pi}\frac{\mu_0}{\mu_0 - \mathring{\imath}\omega}|\widetilde{\nu_i}|^2\,\mathrm{d}\omega \\ &= \vartheta_i^2\mu_0 \begin{cases} \dfrac{e^{-\mu_0|\tau|}}{\beta_i^2 - \mu_0^2} - \dfrac{e^{-\beta_i|\tau|}}{2\beta_i(\beta_i - \mu_0)}, & \text{if } \tau \le 0, \\ \dfrac{e^{-\beta_i|\tau|}}{2\beta_i(\beta_i + \mu_0)}, & \text{if } \tau \ge 0. \end{cases} \end{aligned} \tag{48}$$

This is an asymmetric function. It appears in the cross-correlation between variables X and Y when X is instantaneously affected by an Ornstein–Uhlenbeck noise source, while Y is affected by the same noise source with a delay characterize by rate μ_0. For example, the effect of noise in the synthesis of a protein on its concentration involves such a delay; as a result, the function $A_i(\tau)$ appears in the covariance between π_i and ϕ_i.

The third function is

$$\begin{aligned} S_i(\tau) &:= \int \frac{e^{\mathring{\imath}\omega\tau}}{2\pi}\frac{\mu_0^2}{\mu_0^2 + \omega^2}|\widetilde{\nu_i}|^2\,\mathrm{d}\omega \\ &= \frac{\vartheta_i^2\mu_0^2}{2(\beta_i^2 - \mu_0^2)}\left(\frac{e^{-\mu_0|\tau|}}{\mu_0} - \frac{e^{-\beta_i|\tau|}}{\beta_i}\right). \end{aligned} \tag{49}$$

This is again a symmetric function. It emerges whenever the covariance is taken between variables that are both affected by an Ornstein–Uhlenbeck process with the same delay characterized by rate μ_0. We note that

$$A_i(\tau) + A_i(-\tau) = 2S_i(\tau). \tag{50}$$

Auto-covariances and coefficients of variation

In terms of the functions $B(t)$, $A(t)$, and $S(t)$, the auto-covariances for ϕ_i, π_i, and μ are given by

$$\frac{\chi_{\phi_i\phi_i}(\tau)}{\phi_{0,i}^2} = (1-2\phi_{0,i})S_i(\tau) + \sum_j \phi_{0,j}^2 S_j(\tau), \tag{51}$$

$$\frac{\chi_{\pi_i\pi_i}(\tau)}{\pi_{0,i}^2} = B_i(\tau) + 2C_i^\mu S_i(\tau) + \sum_j \left(C_j^\mu\right)^2 S_j(\tau), \tag{52}$$

$$\frac{\chi_{\mu\mu}(\tau)}{\mu_0^2} = \sum_j \left[C_j^\mu\left(C_j^\mu + 2\phi_{0,j}\right)S_j(\tau) + \phi_{0,j}^2 B_j(\tau)\right]. \tag{53}$$

From these auto-covariances we obtain the CVs η_x:

$$\eta_{\phi_i}^2 = \left(\frac{\mu_0(1-2\phi_{0,i})}{2\beta_i(\beta_i+\mu_0)}\right)\vartheta_i^2 + \sum_j \left(\frac{\mu_0\phi_{0,j}^2}{2\beta_j(\beta_j+\mu_0)}\right)\vartheta_j^2 \tag{54}$$

$$\eta_{\pi_i}^2 = \left(\frac{1}{2\beta_i} + \frac{C_i^\mu\mu_0}{\beta_i(\beta_i+\mu_0)}\right)\vartheta_i^2 + \sum_j \left(\frac{\mu_0\left(C_j^\mu\right)^2}{2\beta_j(\beta_j+\mu_0)}\right)\vartheta_j^2 \tag{55}$$

$$\eta_\mu^2 = \sum_j \left(\frac{C_j^\mu(C_j^\mu+2\phi_{0,j})\mu_0}{2\beta_j(\beta_j+\mu_0)} + \frac{\phi_{j,0}^2}{2\beta_j}\right)\vartheta_j^2 \tag{56}$$

Here we used equation (43) and the special cases $B_i(0) = \vartheta_i^2/(2\beta_i)$ and $A_i(0) = S_i(0) = \vartheta_i^2\mu_0\left[2\beta_i(\beta_i+\mu_0)\right]^{-1}$ derived from equations (47), (48), and (49).

Intrinsic and extrinsic noise

Defining the shorthand

$$S_i := S_i(0) = \frac{\vartheta_i^2\mu_0}{2\beta_i(\beta_i+\mu_0)} = \frac{\mathrm{Var}(N_i)}{\phi_{0,i}^2\mu_0(\mu_0+\beta_i)} \tag{57}$$

as a compound parameter associated to noise source i, where $\mathrm{Var}(N_i)$ is the variance of the Ornstein–Uhlenbeck process N_i, allows us to write equation (54) in the form of equation (14) of the main text, or as:

$$\eta_{\phi_i}^2 = \underbrace{(1-\phi_{0,i})^2 S_i}_{\text{intrinsic}} + \underbrace{\sum_{j\neq i}\phi_{0,j}^2 S_j}_{\text{extrinsic}}. \tag{58}$$

The two terms can be interpreted as intrinsic and extrinsic components of the noise. A discussion of this expression is given in the main text.

To see how the intrinsic and extrinsic components behave as a function of the protein's mean mass fraction $\phi_{0,i}$, we assume that the relative noise amplitude ϑ_i scales as $\phi_{0,i}^{-1/2}$, so that $S_i \sim \phi_{0,i}^{-1}$. Equation (58) then predicts two regimes: at low expression levels, the intrinsic noise dominates and the CV scales as $\phi_{0,i}^{-1/2}$. At high expression levels, the intrinsic component becomes negligible compared to the extrinsic component, and the CV approaches a plateau. This type of scaling has indeed been observed in experiments [51] and has been predicted by other theory as well [16].

Cross-covariances and cross-correlations

The second half of the main text focuses on the analysis of cross-correlations between gene expression and growth. Here we give explicit expressions for the cross-covariances between expression levels $\boldsymbol{\phi}$, protein production rates $\boldsymbol{\pi}$, and the growth rate μ; in conjunction with the CVs from equations (54), (55), and (56), these expressions can be used to to compute the cross-correlation functions.

The cross-covariances are:

$$\frac{\chi_{\phi_i\mu}(\tau)}{\phi_{0,i}\mu_0} = \underbrace{C_i^\mu S_i(\tau)}_{\text{Control}} + \underbrace{\phi_{0,i}A_i(\tau)}_{\text{Autogenic}} \underbrace{- \sum_j \phi_{0,j}\left[C_j^\mu S_j(\tau) + \phi_{0,j}A_j(\tau)\right]}_{\text{Dilution}}. \tag{59}$$

$$\frac{\chi_{\pi_i\mu}(\tau)}{\pi_{0,i}\mu_0} = \underbrace{C_i^\mu A_i(-\tau)}_{\text{Control}} + \underbrace{\phi_{0,i}B_i(\tau)}_{\text{Autogenic}} \underbrace{+ \sum_j C_j^\mu\left[C_j^\mu S_j(\tau) + \phi_{0,j}A_j(\tau)\right]}_{\text{Transmission}}. \tag{60}$$

$$\frac{\chi_{\phi_i\phi_j}(\tau)}{\phi_{0,i}\phi_{0,j}} = -\phi_{0,i}S_i(\tau) - \phi_{0,j}S_j(\tau) + \sum_k \phi_k^2 S_k(\tau). \tag{61}$$

$$\frac{\chi_{\pi_i\pi_j}(\tau)}{\pi_{0,i}\pi_{0,j}} = C_i^\mu A_i(-\tau) + C_j^\mu A_j(\tau) + \sum_k \left(C_k^\mu\right)^2 S_k(\tau). \tag{62}$$

In these expressions we have indicated the four noise modes as defined and illustrated in the main text.

The cross-covariance between expression levels of different proteins $\chi_{\phi_i\phi_j}(\tau)$ is symmetrical. Any asymmetry that may be observed in this function will therefore remain unexplained by our model. Such an asymmetry could be explained by noise sources outside of protein production that act differently on the two proteins, or by gene regulation.

Whereas $\chi_{\phi_i\phi_j}(\tau)$ is fully determined by the expression levels $\boldsymbol{\phi}$ and the noise properties of the proteins, the cross-covariance between protein production rates $\chi_{\pi_i\pi_j}(\tau)$ contains information about the GCCs. Measurements on this function may therefore shed light on the growth-control properties of proteins.

Results on growth-control coefficients

Relation between GCCs and FCCs

As stated in the main text, the growth-control coefficient C_i^μ, flux control coefficient C_i^J, and mean mass fraction $\phi_{0,i}$ of a protein species i are tightly related through

$$C_i^J = C_i^\mu + \phi_{0,i}. \tag{63}$$

Here, we provide a derivation. (See also [47, 56] for a similar derivation and application in an evolutionary context.)

By definition, $\mu_\mathrm{d}(\boldsymbol{n}) := J(\boldsymbol{n})/M(\boldsymbol{n})$; equation (63) then follows from the product rule for differentiation:

$$\begin{aligned}
C_i^J &= \left[\frac{\phi_i}{J(\boldsymbol{\phi})}\frac{\partial J(\boldsymbol{\phi})}{\partial \phi_i}\right]_{\boldsymbol{\phi_0}} \\
&= \frac{\phi_{0,i}}{\mu_\mathrm{d}(\boldsymbol{\phi_0})M(\boldsymbol{\phi_0})}\left[\frac{\partial\left[\mu_\mathrm{d}(\boldsymbol{\phi})M(\boldsymbol{\phi})\right]}{\partial \phi_i}\right]_{\boldsymbol{\phi_0}} \\
&= \frac{\phi_{0,i}}{\mu_\mathrm{d}(\boldsymbol{\phi_0})}\left(\frac{1}{M(\boldsymbol{\phi_0})}\left[\frac{\partial \mu_\mathrm{d}}{\partial \phi_i}\right]_{\boldsymbol{\phi_0}} + \mu_\mathrm{d}(\boldsymbol{\phi_0})\left[\frac{\partial M}{\partial \phi_i}\right]_{\boldsymbol{\phi_0}}\right) \\
&= C_i^\mu + \phi_{0,i}.
\end{aligned} \tag{64}$$

In this derivation, we used that $\partial M(\boldsymbol{\phi})/\partial\phi_i = 1$ and $M(\boldsymbol{\phi_0}) = 1$, which both follow from the definition $M(\boldsymbol{n}) := \sum n_i$.

Sum rules

In this section, we provide derivations of the sum rules for flux control coefficients (FCCs) and growth-control coefficients (GCCs)

In metabolic control analysis [45, 46] , the FCC of enzyme i with respect to a metabolic flux J has been defined as

$$C_i^J := \left[\frac{\phi_i}{J}\frac{\partial J}{\partial \phi_i}\right]_{\boldsymbol{\phi_0}} \tag{65}$$

where ϕ_i is the concentration of enzyme i. A classical result is the sum rule for FCCs:

$$\sum_i C_i^J = 1. \tag{66}$$

This result is ultimately based on the common assumption that metabolic fluxes are *extensive* quantities; that is, for any $\alpha > 0$ and any composition $\boldsymbol{\phi}$ it is assumed that

$$J(\alpha\boldsymbol{\phi}) = \alpha J(\boldsymbol{\phi}). \tag{67}$$

Differentiating both sides of this equation with respect to α and evaluating the result in $\alpha = 1$ yields:

$$\sum_i \phi_i \frac{\partial J}{\partial \phi_i} = J(\boldsymbol{\phi}), \tag{68}$$

where the summation runs over all protein species. Dividing by J directly results in the sum rule of equation (65).

Our definition of growth-control coefficients (GCCs) proceeds analogously, as

$$C_i^\mu := \left[\frac{\phi_i}{\mu_\mathrm{d}}\frac{\partial \mu_\mathrm{d}}{\partial \phi_i}\right]_{\boldsymbol{\phi_0}} \tag{69}$$

For GCCs, however, a different sum rule holds:

$$\sum_i C_i^\mu = 0. \tag{70}$$

The derivation of this result is directly analogous to that for FCCs, except that μ_d is assumed to be *intensive* rather than extensive. That is, for any $\alpha > 0$ and any composition $\boldsymbol{\phi}$ it is assumed that

$$\mu_\mathrm{d}(\alpha\boldsymbol{\phi}) = \mu_\mathrm{d}(\boldsymbol{\phi}). \tag{71}$$

Differentiating both sides with respect to α and evaluating the resulting expression in $\alpha = 1$ now gives

$$\sum_i \phi_i \frac{\partial \mu_\mathrm{d}}{\partial \phi_i} = 0. \tag{72}$$

Dividing by μ_d directly results in the sum rule of equation (70).

Application to the two-protein toy model

In the main text, a toy model is presented in which the cell contains only two protein species, called X and Y. We here derive the mathematical expressions underlying these results.

Cross-correlations

For the two-protein model, the general expressions of equations (45), (59) and (60) evaluate to the following cross-correlations $R_{\phi_Y\mu}(\tau)$ and $R_{\pi_Y\mu}(\tau)$:

$$R_{\phi_Y\mu}(\tau) = \frac{\overbrace{C^\mu_Y S_Y(\tau)}^{\text{Control}} + \overbrace{\phi_{0,Y}A_Y(\tau)}^{\text{Autogenic}} \overbrace{- \phi_{0,Y}\left[C^\mu_Y S_Y(\tau) + \phi_{0,Y}A_Y(\tau)\right] + \phi_{0,X}\left[C^\mu_X S_X(\tau) + \phi_{0,X}A_X(\tau)\right]}^{\text{Dilution}}}{\eta_{\phi_Y}\eta_\mu}. \tag{73}$$

$$R_{\pi_Y\mu}(\tau) = \frac{\overbrace{C^\mu_Y A_Y(-\tau)}^{\text{Control}} + \overbrace{\phi_{0,Y}B_Y(\tau)}^{\text{Autogenic}} + \overbrace{C^\mu_X\left[C^\mu_X S_X(\tau) + \phi_{0,X}A_X(\tau)\right] + C^\mu_Y\left[C^\mu_Y S_Y(\tau) + \phi_{0,Y}A_Y(\tau)\right]}^{\text{Transmission}}}{\eta_{\pi_Y}\eta_\mu}, \tag{74}$$

where the coefficients of variation in the denominators are given by

$$\eta^2_{\phi_Y} = \left(\frac{(1-\phi_{0,Y})^2\mu_0\vartheta^2_Y}{2\beta_Y(\beta_Y+\mu_0)} + \frac{\phi^2_{0,X}\mu_0\vartheta^2_X}{2\beta_X(\beta_X+\mu_0)}\right) \tag{75}$$

$$\eta^2_{\pi_Y} = \left(1 + \frac{\mu_0 C^\mu_Y\left(C^\mu_Y+2\right)}{\beta_Y+\mu_0}\right)\frac{\vartheta^2_Y}{2\beta_Y} + \frac{\mu_0\left(C^\mu_X\right)^2\vartheta^2_X}{2\beta_X(\beta_X+\mu_0)} \tag{76}$$

$$\eta^2_\mu = \left(\frac{\mu_0 C^\mu_X\left(C^\mu_X+2\phi_{0,X}\right)}{\beta_X+\mu_0} + \phi^2_{0,X}\right)\frac{\vartheta^2_X}{2\beta_X} + \left(\frac{\mu_0 C^\mu_Y\left(C^\mu_Y+2\phi_{0,Y}\right)}{\beta_Y+\mu_0} + \phi^2_{0,Y}\right)\frac{\vartheta^2_Y}{2\beta_Y}. \tag{77}$$

The expressions above can be further simplified by inserting the constraints

$$C^\mu_X + C^\mu_Y = 0, \tag{78}$$

$$\phi_{0,X} + \phi_{0,Y} = 1. \tag{79}$$

Regulation in the two-protein model

In deriving the results above, we assumed that $\boldsymbol{f}$ is constant; that is, the synthesis rate of the proteins is not dynamically regulated in response to fluctuations in the protein mass fractions. Generally, if gene regulation is included, analytical results become hard to obtain. The simple case of the two-protein toy model is an exception, as we now show.

Assuming, as before, that $\boldsymbol{f}$ is an intensive quantity, we find the additional constraint (sum rule)

$$C^{f_Y}_X + C^{f_Y}_Y = 0. \tag{80}$$

Furthermore, $\boldsymbol{f}$ denotes the fractional allocation of resources towards each protein species, so that

$$f_X + f_Y = 1. \tag{81}$$

Therefore, the regulation has only one degree of freedom and is thus fully characterized by just one parameter:

$$C^{f_X}_X = C^{f_Y}_Y = -C^{f_Y}_X = -C^{f_X}_Y, \tag{82}$$

which quantifies the auto-regulation of protein X, that of Y, the regulation of Y by X, and *vice versa*.

These constraints allow us to calculate the two-protein cross-correlations analytically. Equation (35) simplifies to

$$\begin{aligned}\frac{\delta\dot{\phi}_Y}{\phi_{0,Y}\mu_0} &= C^{f_Y}_X\frac{\delta\phi_X}{\phi_{0,X}} + C^{f_Y}_Y\frac{\delta\phi_Y}{\phi_{0,Y}} \\ &\quad + (1-\phi_{0,Y})(\nu_Y-\nu_X) - \frac{\delta\phi_Y}{\phi_{0,Y}} \\ &= -C^{f_Y}_Y\frac{-\delta\phi_Y}{1-\phi_{0,Y}} + C^{f_Y}_Y\frac{\delta\phi_Y}{\phi_{0,Y}} \\ &\quad + (1-\phi_{0,Y})(\nu_Y-\nu_X) - \frac{\delta\phi_Y}{\phi_{0,Y}} \\ &= \left(\frac{C^{f_Y}_Y}{1-\phi_{0,Y}} - 1\right)\frac{\delta\phi_Y}{\phi_{0,Y}} \\ &\quad + (1-\phi_{0,Y})(\nu_Y-\nu_X).\end{aligned} \tag{83}$$

The Fourier transform then reads

$$\widetilde{\frac{\delta\phi_Y}{\phi_{0,Y}}} = (1-\phi_{0,Y})\left(\frac{\mu_0}{\mu_Y + \mathring{\imath}\omega}\right)\left(\widetilde{\nu_Y} - \widetilde{\nu_X}\right). \tag{84}$$

This equation differs only slightly from equation (36): apart from a constant prefactor $(1-\phi_{0,Y})$, a term μ_0 in the denominator is replaced by

$$\mu_Y = \mu_0\left(1 - \frac{C^{f_Y}_Y}{1-\phi_{0,Y}}\right). \tag{85}$$

Because equations (36) and (84) have the same form, their solutions are analogous.

To illustrate the effect of regulation in this toy model, the expression–growth cross-correlation function is plotted in Fig S2A, for different values of $C_{\mathrm{Y}}^{f_{\mathrm{Y}}}$. Positive auto-regulation widens the cross-correlation function, indicating increased auto-correlation timescales; negative auto-regulation narrows it, indicating decreased auto-correlation timescales. Without regulation, dilution by growth quenches fluctuations in the concentration ϕ_{Y} with the associated time scale μ_0^{-1}. In the presence of regulation this time scale is adjusted to μ_{Y}^{-1}. This directly determines the time scales of the cross-correlations.

Furthermore, regulation affects the CV of the expression of both X and Y, because the term μ_0 in the denominator of equation (75) is replaced by to μ_{Y}. This is shown in Fig S2B. If $C_{\mathrm{Y}}^{f_{\mathrm{Y}}}$ is negative (negative auto-regulation), fluctuations are actively quenched; the variance of ϕ_{Y} is therefore reduced. Conversely, if $C_{\mathrm{Y}}^{f_{\mathrm{Y}}}$ is positive (positive auto-regulation) fluctuations are amplified; the variance in ϕ_{Y} is therefore increased.

Stochastic simulation of the two protein model

To test the analytical results on the two-protein toy model, we used the Euler–Maruyama method to propagate the system of stochastic differential equations and thus calculated the cross-correlations numerically. These simulations are based on the non-linear equations (4), (13) and (14), and a non-linear function $\mu_{\mathrm{d}}(\phi)$ was used. As such, they demonstrate that, provided $\mu_{\mathrm{d}}(\phi)$ is sufficiently smooth, the approximations used in the derivation of the linearized equations are appropriate. The results are included in Fig 3AB.

In this simulation we chose the parameters of the non-linear system such as to match the parameters used for the plots in Fig 3AB. Specifically, we specified the allocation coefficient as $f_{\mathrm{Y}} = 0.33$, and the parameters of the noise sources as $\theta_{\mathrm{Y}} = 0.5$, $\theta_{\mathrm{X}} = 0.5$, $\beta_{\mathrm{Y}} = 4$/h, and $\beta_{\mathrm{X}} = 4$/h, as in Fig 3AB. For the the deterministic part of the growth rate we chose $\mu_{\mathrm{d}}(\phi) := (a_{\mathrm{X}}\phi_{\mathrm{X}} + a_{\mathrm{Y}}\phi_{\mathrm{Y}})/(\phi_{\mathrm{X}} + \phi_{\mathrm{Y}})$, with $a_{\mathrm{X}} = 0.63$/h and $a_{\mathrm{Y}} = 1.37$/h, such that $\mu_0 = \mu_{\mathrm{d}}(\boldsymbol{\phi_0}) \approx \mu_{\mathrm{d}}(\boldsymbol{f}) = 1$/h and the control coefficients would approximate the ones in Fig 3 ($C_Y^{\mu} = -C_X^{\mu} = 0.25$).

We propagated the dynamical equations in discrete time steps Δt by repeating the following calculations. (Here, the index i is either X or Y.)

$$\begin{cases} \mu_{\mathrm{d}}(t) = (a_{\mathrm{X}}\phi_{\mathrm{X}}(t) + a_{\mathrm{Y}}\phi_{\mathrm{Y}}(t))/(\phi_{\mathrm{X}}(t) + \phi_{\mathrm{Y}}(t)), \\ \pi_i(t) = f_i\, \mu_{\mathrm{d}}(t) + N_i(t), \\ \mu(t) = \mu_{\mathrm{d}}(t) + N_{\mathrm{X}}(t) + N_{\mathrm{Y}}(t), \\ \phi_i(t+\Delta t) = \phi_i(t)\,(1 - \Delta t \mu(t)) + \Delta t\, \pi_i(t), \\ N_i(t+\Delta t) = N(t)\,(1-\beta_i \Delta t) + \sqrt{\Delta t}\, \theta_i \mathrm{d}W(t). \end{cases} \tag{86}$$

In the last line, $\mathrm{d}W(t)$ is random variable drawn from a standard normal distribution each time step. We ran the simulation for roughly 30.000 doubling times, using time steps $\Delta t = 10^{-3}$.

After the simulation, we measured ϕ_{Y} to test whether $f_{\mathrm{Y}} \approx \phi_{\mathrm{Y}}$ as is assumed in section 2.5. Indeed, f_{Y} did not differ from ϕ_{Y} by more than 5%. We next verified that $\mu_0 := \mu_{\mathrm{d}}(\boldsymbol{\phi_0}) \approx 0.993$/h was close to 1/h and that $C_{\mathrm{Y}}^{\mu} = \phi_{0,\mathrm{Y}}(a_{\mathrm{Y}}/\mu_0 - 1) \approx 0.249$ was close to 0.25, as intended.

Application to the many-protein model

As described in the main text, we applied the modeling framework to model a full cell, containing 1021 protein species in total. We here provide the mathematical details of this model. First we explain how the operon construct was represented. Then we describe our parameter choices and how they were informed by previously published experimental data.

GFP reporter fused to the *lac* operon

As mentioned in the main text, Kiviet *et al.* performed experiments in which the expression levels of the genes *lacY* and *lacZ* were reported by a green fluorescent protein (GFP), whose gene was transcriptionally fused to the *lac* operon [5]. Thus, the GFP signal was used as a proxy for the concentration of the *lac* proteins LacY and LacZ. To include this construct in the model, we assumed that the synthesis noise of each protein, introduced by transcription of the operon and the translation of its genes, can be linearly decomposed into two components. The first component reflects noise originating from processes that similarly affect all genes on the operon, adding to the correlation between their expression levels. In particular, this includes fluctuations in the copy number of the mRNA transcript due to transcriptional noise. The second component reflects noise originating from processes that affect each gene independently, including all post-transcriptional processes.

Formally, we included noise sources N_{Y}, N_{Z}, and N_{G}, respectively acting on LacY, LacZ, and GFP synthesis only, as well an additional noise source N_{O} that simultaneously affects the synthesis of all of these proteins. That is, equation (13) was modified to:

$$\pi_i = f_i \mu_{\mathrm{d}}(\boldsymbol{\phi_0}) + N_i + I_i^{\mathrm{O}} \frac{\pi_{0,i}}{\pi_{0,\mathrm{O}}} N_{\mathrm{O}}. \tag{87}$$

Here, I_i^{O} is an indicator function that specifies whether protein i is encoded on the *lac* operon: $I_i^{\mathrm{O}} = 1$ for $i \in \mathrm{O} := \{\mathrm{Y}, \mathrm{Z}, \mathrm{G}\}$ and $I_i^{\mathrm{O}} = 0$ otherwise.

As before, all noise sources are mutually independent Ornstein–Uhlenbeck processes with zero mean. For simplicity, we incorporated the operon noise such that it affects the three genes in proportion with their average production rate. In the limit that $\nu_i = 0$ for $i \in \mathrm{O}$ and $N_{\mathrm{O}} \neq 0$, the three proteins behave as a single protein O, with the same scaling $N_j = \pi_{0,j}\nu_j$ as the other genes.

After linearization (cf. equation (25)) we arrive at

$$\frac{\delta\pi_i}{\pi_{0,i}} = \sum_j C_j^{\mu} \frac{\delta\phi_j}{\phi_{0,j}} + \nu_i + I_i^{\mathrm{O}} \nu_{\mathrm{O}}. \tag{88}$$

Here, the summation runs over all 1021 proteins, including Y, Z, and G. Moreover, we have defined $\nu_O := N_{\mathrm{O}}/\pi_{0,\mathrm{O}} = N_O/(\pi_{0,\mathrm{Y}} + \pi_{0,\mathrm{Z}} + \pi_{0,\mathrm{G}})$. To obtain the cross-covariances, the analysis now proceeds exactly as before. Using the shorthands $\phi_{0,\mathrm{O}} := \phi_{0,\mathrm{Y}} + \phi_{0,\mathrm{Z}} + \phi_{0,\mathrm{G}}$ and $C_{\mathrm{O}}^{\mu} := C_{\mathrm{Y}}^{\mu} + C_{\mathrm{Z}}^{\mu} + C_{\mathrm{G}}^{\mu}$, the resulting (cross-)covariances are given by

$$\frac{\chi_{\phi_i\phi_i}(\tau)}{\phi_{0,i}^2} = (1 - 2\phi_{0,i}) S_i(\tau) + I_i^{\mathrm{O}} (1 - 2\phi_{0,\mathrm{O}}) S_{\mathrm{O}}(\tau) + \sum_j \phi_{0,j}^2 S_j(\tau); \tag{89}$$

$$\frac{\chi_{\pi_i\pi_i}(\tau)}{\pi_{0,i}^2} = B_i(\tau) + 2C_i^{\mu} S_i(\tau) + I_i^{\mathrm{O}} \left[B_{\mathrm{O}}(\tau) + 2C_{\mathrm{O}}^{\mu} S_{\mathrm{O}}(\tau)\right] + \sum_j \left(C_j^{\mu}\right)^2 S_j(\tau); \tag{90}$$

$$\frac{\chi_{\mu\mu}(\tau)}{\mu_0^2} = \sum_j \left\{\phi_j^2 B_j(\tau) + \left[\left(C_j^{\mu}\right)^2 + 2\phi_{0,j} C_j^{\mu}\right] S_j(\tau)\right\}; \tag{91}$$

$$\eta_{\phi_i}^2 = (1 - 2\phi_{0,i}) \frac{\mu_0 \vartheta_i^2}{2\beta_i(\beta_i + \mu_0)} + I_i^{\mathrm{O}} (1 - 2\phi_{\mathrm{O}}) \frac{\mu_0 \vartheta_{\mathrm{O}}^2}{2\beta_{\mathrm{O}}(\beta_{\mathrm{O}} + \mu_0)} + \sum_j \phi_{0,j}^2 \frac{\mu_0 \vartheta_j^2}{2\beta_j(\beta_j + \mu_0)}; \tag{92}$$

$$\frac{\chi_{\phi_i\mu}(\tau)}{\phi_{0,i}\mu_0} = C_i^{\mu} S_i(\tau) + \phi_{0,i} A_i(\tau) + I_i^{\mathrm{O}} \left[C_{\mathrm{O}}^{\mu} S_{\mathrm{O}}(\tau) + \phi_{0,\mathrm{O}} A_{\mathrm{O}}(\tau)\right] - \sum_j \phi_{0,j} \left[C_j^{\mu} S_j(\tau) + \phi_{0,j} A_j(\tau)\right]; \tag{93}$$

$$\frac{\chi_{\pi_i\mu}(\tau)}{\pi_{0,i}\mu_0} = \phi_{0,i} B_i(\tau) + C_i^{\mu} A_i(-\tau) + I_i^{\mathrm{O}} \left[\phi_{0,\mathrm{O}} B_{\mathrm{O}}(\tau) + C_{\mathrm{O}}^{\mu} A_{\mathrm{O}}(-\tau)\right] + \sum_j C_j^{\mu} \left[C_j^{\mu} S_j(\tau) + \phi_{0,j} A_j(\tau)\right]. \tag{94}$$

The index j of the summations now runs over all proteins as well as the operon $j = \mathrm{O}$, so that all noise sources are included.

Parametrization

In order to simulate a whole cell, we had to specify four parameters for each protein i, namely its abundance $\phi_{0,i}$, its control coefficient C_i^{μ}, and its noise properties ϑ_i and β_i. In addition, the mean growth rate μ_0 had to be set for each of the three growth states presented in the main text (low, intermediate, and high).

Monod curve

Under the experimental conditions, the mean growth rate μ_0 follows an empirical Monod curve $\mu_{\mathrm{M}}(\phi_{0,\mathrm{O}})$ as a function of the mean expression level $\phi_{0,\mathrm{O}}$ of the *lac* operon [5]. To ensure that our parameters respect this relation, we constrain them with the Monod curve shown in Fig 4B,

$$\mu_{\mathrm{M}}(\phi_{0,\mathrm{O}}) := \mu_{\max} \frac{\phi_{0,\mathrm{O}}}{\phi_{0,\mathrm{O}} + \phi_{\mathrm{half}}}, \tag{95}$$

which is defined by a maximal growth rate of $\mu_{\max} = 0.8\ \mathrm{h}^{-1}$ and $\phi_{\mathrm{half}} = 0.005$. The parameter ϕ_{half} was taken clearly larger than the abundance of an average protein, since transporter proteins are highly expressed in growth media where they are utilized [29].

Choosing the average growth rate μ_0

The average growth rates in the three growth states were chosen in rough agreement with the experiments as $\mu_0 = 0.19$, 0.45, and 0.75 h^{-1}.

Choosing reversion rates β

For simplicity, we assumed that the reversion rates β_i of all noise sources were identical and considerably larger than the average growth rate μ_0; that is, we set $\beta_i = \beta := 4\mu_{\max}$ for all i.

Choosing ϕ_0 and ϑ based on experimental data

Several experimental datasets are available that provide cell-wide estimates of *E. coli* protein abundances [28, 29, 51, 53]. In particular, Taniguchi *et al.* have measured the means and variances of 1018 protein abundances [51]. We used these measurements to fix the values of $\phi_{0,i}$ and ϑ_i for all proteins i in our model, as we now explain.

First we determined the average mass fractions $\boldsymbol{\phi_0}$. Given the mean growth rate μ_0 for a given growth condition, the average mass fraction of the operon, $\phi_{0,\mathrm{O}}$, follows directly from the Monod curve described above. We then assumed

equal properties for the proteins on the operon, i.e. $\phi_{0,\mathrm{G}} = \phi_{0,\mathrm{Y}} = \phi_{0,\mathrm{Z}} = \phi_{0,\mathrm{O}}/3$ and $\vartheta_{0,\mathrm{G}} = \vartheta_{0,\mathrm{Y}} = \vartheta_{0,\mathrm{Z}}$. We denote the sum of all measured protein abundances as m, and the mass of the whole cell including the *lac* operon as M. Then $M = m/(1-\phi_{0,\mathrm{O}})$, and the average mass fraction of protein i can be calculated by dividing the measured abundance by M.

Next, we calculated the values of the noise amplitudes $\boldsymbol{\vartheta}$. Equation (92) shows that the squared coefficient of variation of any given protein species is a linear function of the squared noise amplitudes of all noise sources. Conversely, imposing the measured variances of all protein species results in a linear system of equations, the solution of which uniquely determines the ϑ_i^2 (for given μ_0, $\boldsymbol{\phi_0}$ and β).

To do so explicitly, we assigned all proteins an index in the range 1 to $K+3$, where K is the number of proteins that are not encoded on the *lac* operon (in this case $K = 1018$); proteins G, Y and Z were assigned indices 1 to 3, respectively. We set the amplitude of the operon noise source to $\vartheta_\mathrm{O} = \alpha\vartheta_\mathrm{G} = \alpha\vartheta_\mathrm{Y} = \alpha\vartheta_\mathrm{Z}$, where α defines the ratio of the amplitude of ν_O to the amplitudes of ν_G, ν_Y, and ν_Z .We used $\alpha = 1.5$ to obtain the results in the main text (Figure 4). This procedure allowed us to rewrite equation (92) in $((K+3)\times(K+3)$ dimensional) matrix form:

$$\eta_{\boldsymbol{\phi}}^2 = \boldsymbol{T}\,\boldsymbol{\vartheta^2}, \tag{96}$$

where the vector $\boldsymbol{\vartheta^2}$ contains the squared noise amplitudes, $\boldsymbol{\eta_\phi^2}$ is the vector of squared CVs, and the elements of matrix $\boldsymbol{T}$ are given by

$$T_{ij} = \frac{\mu_0}{2\beta(\beta+\mu_0)}\times \begin{cases} (1-\phi_{0,\mathrm{G}})^2 + (1-\phi_{0,\mathrm{O}})^2\alpha^2 & \text{if } i=j=1. \\ \phi_{0,\mathrm{G}}^2 + (1-\phi_{0,\mathrm{O}})^2\alpha^2 & \text{if } j=1\neq i\leq 3, \\ \phi_{0,\mathrm{G}}^2 + \phi_{0,\mathrm{O}}^2\alpha^2 & \text{if } j=1, i>3, \\ \phi_{0,j}^2 & \text{if } i\neq j\neq 1, \\ (1-\phi_{0,j})^2 & \text{if } i=j\neq 1, \end{cases} \tag{97}$$

Lastly, $\boldsymbol{\vartheta^2}$ was obtained by numerically inverting equation (96).

Choosing the growth-control coefficients

Large-scale measurements of growth-control coefficients are not readily available. Therefore, we randomly sampled the GCCs using the following heuristic. First, we randomly assigned proteins from the database measured by Taniguchi *et al.* to the non-metabolic H sector, until the total H sector size was $\sim$ 25% of the sum of measured protein abundances m. The size of this non-metabolic mass fraction has been directly estimated as 25% by O'Brien *et al.* [48]; it has been described earlier by Hui *et al.* as part of a growth-rate independent proteome fraction that amounts in total to about 60% of the total protein mass [28]. For these H proteins, we set $C_i^\mu = -\phi_i$ (note that ϕ_i partly depends on the expression level of the *lac* operon, as described earlier), in line with equation (11) from the main text.

Second, the GCCs of the *lac*-operon proteins Y, Z, and G were chosen in agreement with the Monod curve discussed above. We next used the derivative of the Monod curve $\mu_\mathrm{M}(\phi_{0,\mathrm{O}})$ to estimate C_O^μ:

$$C_\mathrm{O}^\mu := \left[\frac{\phi_\mathrm{O}}{\mu_\mathrm{d}}\frac{\partial\mu_\mathrm{d}}{\partial\phi_\mathrm{O}}\right]_{\boldsymbol{\phi_0}} \approx \frac{\phi_{0,\mathrm{O}}}{\mu_\mathrm{M}}\frac{\mathrm{d}\mu_\mathrm{M}}{\mathrm{d}\phi_{0,\mathrm{O}}} = \frac{\phi_\mathrm{half}}{\phi_{0,\mathrm{O}}+\phi_\mathrm{half}}. \tag{98}$$

Here we implicitly assumed that the operon as a whole is never so strongly over-expressed as to have a negative control on the growth rate.

Lastly, we chose $C_\mathrm{Y}^\mu = C_\mathrm{Z}^\mu = (C_\mathrm{O}^\mu - C_\mathrm{G}^\mu)/2$ for simplicity.

Third, for the remaining proteins (which are neither in the H sector nor encoded on the *lac* operon), we assumed that the GCCs tend to scale with their abundance, and enforced the sum rule. In practice, we drew a uniformly distributed random number $x_i \in [0,1]$ for each protein that was not in the H sector and set

$$C_i^\mu = \frac{x_i\phi_{0,i}}{\sum_{j\notin\mathrm{H}} x_j\phi_{0,j}}\left(\phi_\mathrm{H} - C_\mathrm{O}^\mu\right). \tag{99}$$

As in the main text, ϕ_H is the mass fraction of all H proteins combined. To determine the robustness of our results with respect to the sampling scheme, we also tried different GCC distributions, for example using exponentially distributed variables x_i. This yielded visually indistinguishable results.

Alternative choice for abundances and variances

Although the data set obtained by Taniguchi *et al.* [51] is unique to report both abundances and variances for each measured protein, it suffers from a systematic underestimation of the protein masses [29, 53]. We therefore also estimated the protein abundances from another proteomics dataset, using a phenomenological model to calculate the variances in the absence of explicit measurements.

In this case, the abundances $\phi_{0,i}$ were taken from a dataset obtained from Arike *et al.* [53]. Given that the mean copy number of protein i is n_i, a theoretical minimum can be calculated for its variance; it is given by $n_i^2\sigma_\mathrm{ab} + n_i\beta_W$, with $\sigma_\mathrm{ab} = 0.025$ and $\beta_W = 450$ [68]. The original paper reported variances that were typically higher than this lower limit [68]. To empirically match these results, we chose

$$\mathrm{Var}_i = n_i^2\sigma_\mathrm{ab} + n_i(\beta_W + x_i), \tag{100}$$

with x_i an exponentially distributed random variable with mean 200.

The resulting cross-correlations are plotted in Fig S3. They lead to the same qualitative conclusions as the analysis based on the data from Taniguchi *et al.*

Alternative model: Noise in the allocation of the flux

Throughout this document we have considered noise sources that act on each protein-synthesis rate independently. Alternatively, one could hypothesize that the observed fluctuations in protein concentrations instead originate from noise on the allocation of the available metabolic flux. Especially when translation is a highly rate-limiting step in protein production, so that mRNAs compete for a limited capacity of ribosomes, a stochastic increase in the synthesis of a given protein species may necessarily go at the expense of the synthesis rates of other protein species. In this section, we therefore explore a model in which such "allocation noise" is dominant instead of the pure "production noise" studied above.

In the modified model, each protein species is allotted a fluctuating fraction $f_i(t)$ of the total metabolic flux, with the additional constraint that $\sum_i f_i(t) = 1$ at all times. We end up with an adjusted version of equation (13):

$$\pi_i := f_i(t)\mu_{\mathrm{d}}(\phi) \tag{101}$$

with

$$f_i(t) = \frac{f_{0,i} + N_i}{\sum_j f_{0,j} + N_j} = \frac{f_{0,i} + N_i}{1 + \sum_j N_j}. \tag{102}$$

Here,the N_i are again independent Ornstein–Uhlenbeck processes. This also results in an adjusted version of equation (14):

$$\mu = \mu_{\mathrm{d}}(\phi). \tag{103}$$

As before, in the small-noise limit μ_{d} can be linearized around the mean composition $\phi_{\mathbf{0}}$:

$$\mu_{\mathrm{d}}(\phi) := \mu_0\left(1 + \sum_j C^{\mu}_j\left(\frac{\phi_j}{f_{0,j}} - 1\right)\right), \tag{104}$$

where we assumed that $\phi_{0,i} \approx f_{0,i}$, as previously.

Unfortunately, production rates as defined in equation (101) are intrinsically correlated and therefore analytical expressions can not be obtained within our current framework. To gain insight in the cross-correlations generated by this system, we therefore numerically integrated equations (101) and (104) for a model cell containing 40 proteins with randomly sampled parameters (see next section for details about parameters and the simulation method). The cross-correlations obtained for one of the proteins (protein 1) are shown in Fig S5A.

A striking observation is that the ϕ–μ cross correlations of the model with "allocation noise" (Fig S5A and C) are always perfectly symmetrical, with their peak at zero delay ($\tau = 0$).

In itself, the presence of a symmetric mode is no surprise. Indeed, an increase in a protein species' synthesis rate, affects—with a delay—its concentration, and this in turn affects the growth rate, in proportion with the protein's GCC. Therefore, a symmetrical control mode is to be expected.

However, when noise acts dominantly on the allocation of the flux, the growth rate is not instantly affected by fluctuating production rates. Therefore, there is now pathway by which noise in a protein's production rate instantly affects the growth rate, and then—after a delay—the protein concentrations (either by dilution or by direct production). This removes the asymmetric autogenic mode that caused the asymmetry in the ϕ–μ cross-correlation as well as the symmetric mode in the ϕ–μ cross-correlation in the "production noise" model (see also Fig 3C–F of the main text).

To check the the symmetric ϕ–μ cross-correlation is indeed a typical feature of the allocation noise model and not just a feature of the particular cell sampled, we analytically calculated cross-correlations for a similar cell (*i.e.* same protein variances, same mean protein abundances, same GGCs), but now according to the "production noise" model (*i.e.*, production rates according to equation (13)). The resulting cross-correlations are shown in Fig S5B and show clear asymmetry in the ϕ–μ cross-correlation (demonstrated in Fig S5C).

Since the measured cross-correlations by Kiviet *et al.* showed clear asymmetrical features as well [5]—which in fact prompted them to propose a "common-noise" source—we conclude that noise on allocation alone cannot explain the observed cross-correlations.

Simulations of the "allocation-noise" model

We here provide details on the simulations discussed in the previous section and presented in Fig S5.

We simulated a cell with 40 proteins. To choose their mean protein fractions, we drew uniformly distributed numbers ($x_j \sim \mathcal{U}[0,1]$, where $\mathcal{U}[0,1]$ is the uniform distribution over the interval $[0,1]$) and normalized them to obtain protein fractions, *i.e.*, $\phi_{i,0} = f_{i,0} = x_i / \sum x_j$. Approximately 60% of the proteome was assumed to be metabolic. For the $\approx$ 40% non-metabolic proteins, we set $C^{\mu}_h = -\phi_{h,0}$; for the remaining proteins we sampled GCCs according to a uniform distribution, scaled with the protein's mean fraction, under the constraint of the sum rule. That is: for any metabolic protein i, draw an $x_i \sim \mathcal{U}[0,1]$, and subsequently set $C^{\mu}_i = \left(\sum_{h\in H}\phi_j\right)(x_i\phi_i) / \left(\sum_{j\notin H} x_j\phi_j\right)$. We also sampled the noise amplitudes $\boldsymbol{\theta}_{\mathrm{AN}}$ (here the label "AN" stands for "allocation noise") uniformly and then scaled them with $\sqrt{f_{i,0}}$, *i.e.*, $\theta_{\mathrm{AN},i} \sim \left(0.1\sqrt{f_{i,0}}\right)\mathcal{U}[0,1]$.

First we numerically integrated Eq.(101)-(104) using the numerical integration scheme below to calculate the cross-correlation for the allocation-noise model.

$$\boldsymbol{\phi}(t+\Delta t) = \boldsymbol{\phi}(t)\left(1 - \Delta t\sum_i \pi_i(t)\right) + \Delta t\,\boldsymbol{\pi}(t), \tag{105}$$

$$\boldsymbol{N}(t+\Delta t) = \boldsymbol{N}(t)\,(1 - \Delta t\,\beta) + \sqrt{\Delta t}\boldsymbol{\theta}_{\mathrm{AN}} * \mathrm{d}W(t), \tag{106}$$

$$\boldsymbol{\pi}(t+\Delta t) = \mu_0 \left(\frac{\boldsymbol{f}_0 + \boldsymbol{N}(t+\Delta t)}{1 + \sum_i N_i(t+\Delta t)} \right) \left(1 + \sum_i C_i^{\mu} \left(\frac{\phi_i(t+\Delta t)}{f_{0,i}} - 1 \right) \right) \tag{107}$$

Here, $*$ denotes element-wise multiplication.

To define a similar cell in the "production-noise" model, we first measured $\boldsymbol{\eta}_{\phi,\mathrm{AN}}$, in the "allocation noise" simulation and used a method similar to equation (96) to calculate which noise amplitudes $\boldsymbol{\theta}_{\mathrm{PN}}$ (PN stands for "production noise") would create the same variances in the "production-noise" model:

$$\boldsymbol{\vartheta}^2_{\mathrm{PN}} = (T_{\mathrm{PN}})^{-1}\, \boldsymbol{\eta}^2_{\phi,\mathrm{AN}}$$

With the matrix T_{PN} similar to equation (97):

$$(T_{\mathrm{PN}})_{ij} = \frac{\mu_0}{2\beta(\beta+\mu 0)} \begin{cases} (1-f_{i,0})^2, & \text{if } i=j, \\ f_{i,0}^2, & \text{else.} \end{cases}$$
