# Supplementary figures and images for: Noise propagation in an integrated model of bacterial gene expression and growth
Source: PLoS Comput Biol. 2018 Oct 5;14(10):e1006386. doi: 10.1371/journal.pcbi.1006386 (PMC6192656; doi:10.1371/journal.pcbi.1006386)

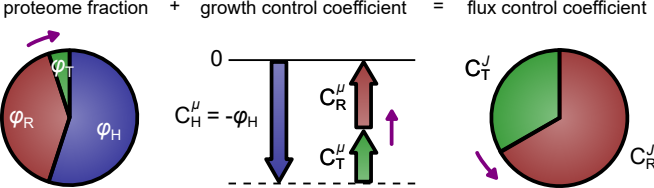

Supplement: S1 Fig — Pictured is the relation between flux-control coefficients CiJ, growth-control coefficients Ciμ, and proteomic mass fractions ϕi for a cell containing just three protein species T, R, and H. Proteins H do not contribute to the global metabolic flux, so that CHJ=0. Purple arrows indicates the effect of a reduction in ϕT in favor of ϕR, which increases the growth control possessed by T. (PDF) [file pcbi.1006386.s002.pdf]

**A** $\phi_Y - \mu$  cross-correlation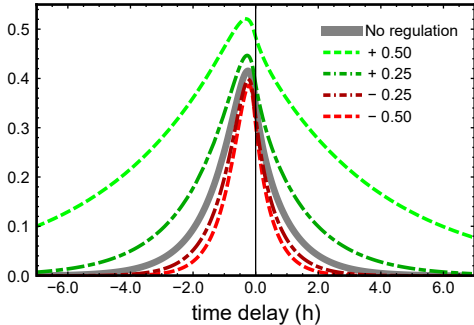**B**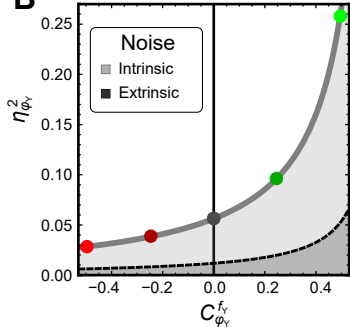

Supplement: S2 Fig — (A) Cross-correlations between the expression level ϕY of protein Y and growth rate μ in the two-protein model, for varying levels of positive (green) and negative (red) auto-regulation, as quantified by the regulatory control coefficient CYfY. The curve plotted in gray is based on CYfY=0 (no auto-regulation), and all other parameters are chosen as in Fig 3A; therefore the gray curve corresponds to the gray curve of Fig 3A. (B) Analytical solution of the coefficient of variation of the concentration of protein Y in the two-protein model, under varying levels of auto-regulation. The intrinsic and extrinsic noise components are indicated by the two shades of gray. The colored circles indicate the parameter choices belonging to the corresponding curves of panel A. (PDF) [file pcbi.1006386.s003.pdf]

**A**

negative control

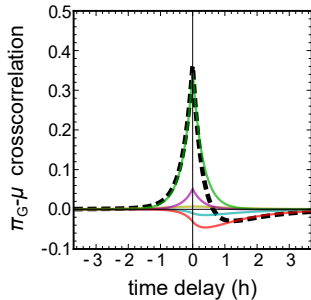**B**

dominant reporter

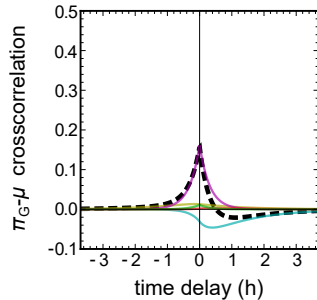**C**

positive control

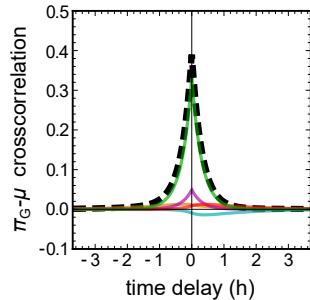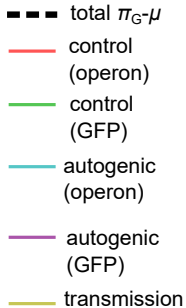

Supplement: S4 Fig — This figure shows the cross-correlations between GFP abundance and growth rate for the same parameters whose concentration–growth cross-correlations were studied in Fig 5. (A) The operon has a negative growth-control coefficient (cf. Fig 5A). (B) Fluctuations in GFP are dominated by its private noise source NG, and are therefore largely decoupled from the fluctuations in the rest of the lac operon (cf. Fig 5B). (C) Highly symmetrical πG–μ cross-correlation despite a (slightly) positive control of the operon, which is masked by the negative control carried by the reporter protein as well as by the asymmetrical transmission mode. (PDF) [file pcbi.1006386.s005.pdf]

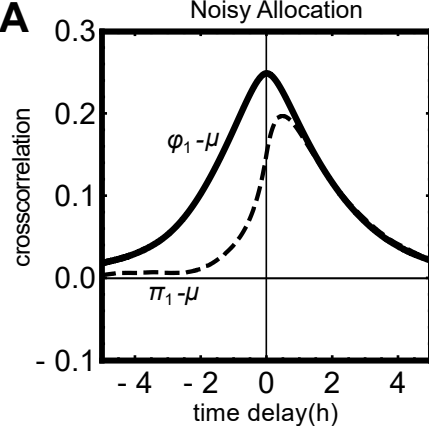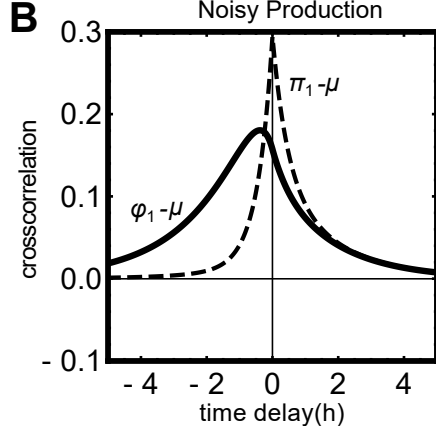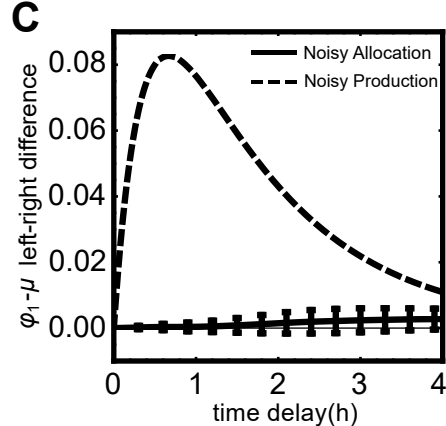

Supplement: S5 Fig — (A) Simulations of an alternative model in which the noise sources act on the allocation of the flux rather than on each protein synthesis rate independently. Shown are the ϕ1–μ (solid line) and π1–μ (dashed line) cross-correlations of protein 1 in a cell containing 40 protein species with arbitrary parameters (see S1 Text pp. 12–13 for more details about the simulation). Here, ϕ0,1 = 0.027 and C1μ=0.022. (B) Analytical results for the ϕ1–μ (solid line) and π1–μ (dashed line) cross-correlations of the same cell, but where noise again acts on each protein synthesis rate independently. The amplitudes of the noise sources were adjusted such that the variances of all protein species were identical to those in panel (A). (C) The asymmetry of the ϕ1–μ cross-correlations R(ϕ1, μ)(τ) shown in panels A (solid line) and B (dashed line), quantified as Rϕ1μ(−τ) − Rϕ1μ(τ). In order to estimate error bars, we repeated the “noisy allocation” simulation of the exact same cell 10 times; error bars indicate the standard error of the mean. Note that, for the “noisy allocation” model, zero lies within the narrow error bars, showing that the cross-correlation is highly symmetric and therefore shows no evidence for an asymmetric mode akin to the autogenic or common-noise mode. (PDF) [file pcbi.1006386.s006.pdf]
