# Supplementary material for: Noise propagation in an integrated model of bacterial gene expression and growth
Source: PLoS Comput Biol. 2018 Oct 5;14(10):e1006386. doi: 10.1371/journal.pcbi.1006386 (PMC6192656; doi:10.1371/journal.pcbi.1006386)
Supplement: S3 Fig — Analysis of the model with protein abundances taken from Arike et al. [53], and variances sampled from a phenomenological noise model (see [16] and S1 Text, Eq. (100) on p. 11). This figure is equivalent to Fig 4, except that it is based on different protein abundances and variances. (A) Distribution of protein abundances and variances. Each gray dot represents a protein; the black points indicate the abundance and variance of the GFP reporter under the three growth condition (equivalent to Fig 4C). (B)–(D) Growth rate cross-correlations between GFP concentration and growth rate (top panels) and GFP synthesis rate and growth rate (bottom panels), for the three growth conditions (equivalent to Fig 4D, 4E and 4F). (PDF) [file pcbi.1006386.s004.pdf]

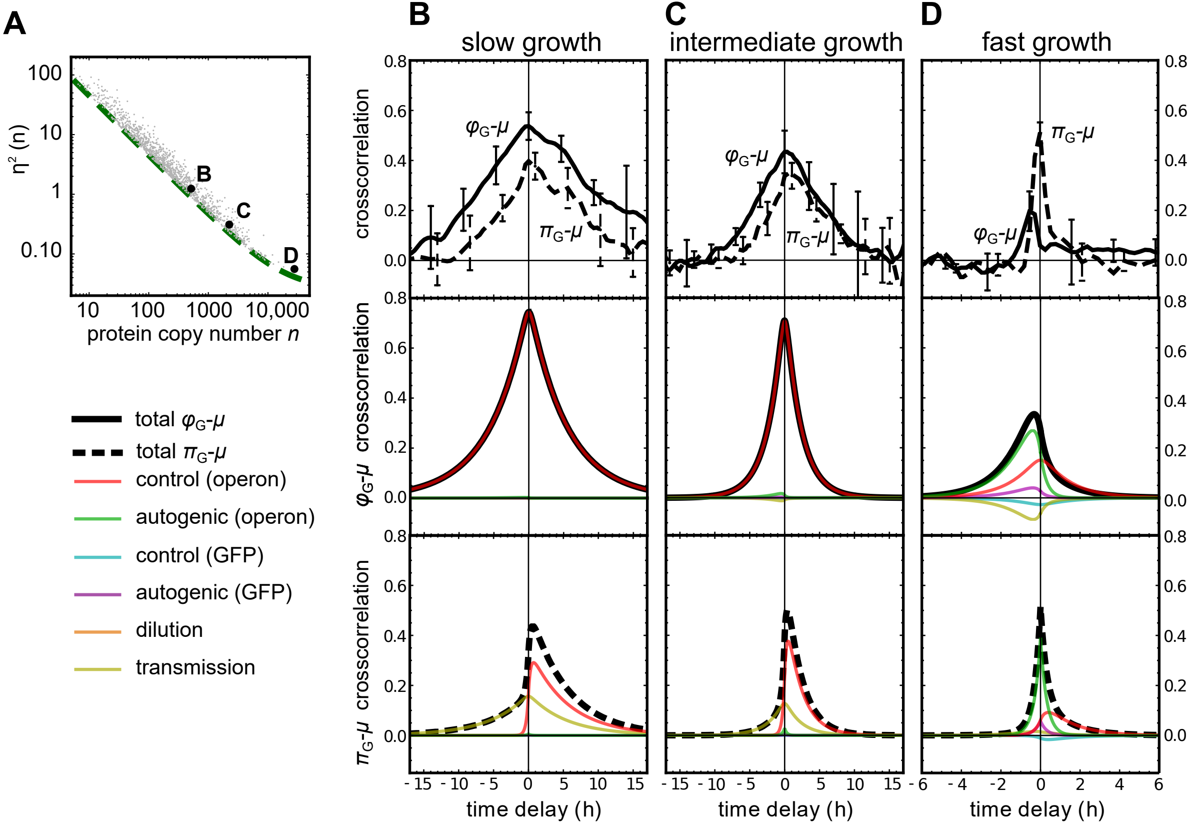
A
B
C
D
slow growth
intermediate growth
fast growth
η^2 (n)
protein copy number *n*
crosscorrelation
φ_G-μ crosscorrelation
π_G-μ crosscorrelation
φ_G-μ
π_G-μ
total φ_G-μ
total π_G-μ
control (operon)
autogenic (operon)
control (GFP)
autogenic (GFP)
dilution
transmission
time delay (h)
